# Supplementary material for: Insights into Circovirus Host Range from the Genomic Fossil Record
Source: J Virol. 2018 Jul 31;92(16):e00145-18. doi: 10.1128/JVI.00145-18 (PMC6069186; doi:10.1128/JVI.00145-18)
Supplement: Supplemental material [file supp_92_16_e00145-18__index.html]

Insights into Circovirus Host Range from the Genomic Fossil Record — Supplemental material 

# Insights into Circovirus Host Range from the Genomic Fossil Record

## Supplemental material

- Supplemental file 1 -

  Fig. S1 (Maximum likelihood phylogeny tree reconstructed from an alignment of replication-associated protein (Rep) sequences.)

  Table S1 (Circovirus and cyclovirus reference sequences.)

  References

  PDF, 160K
- Supplemental file 2 -

  Table S2 (Details of WGS assemblies screened in this study.)

  XLSX, 59K
- Supplemental file 3 -

  Table S3 (Details of all circovirus sequences identified in this study.)

  XLSX, 85K
