# Supplementary material for: Insights into Circovirus Host Range from the Genomic Fossil Record
Source: J Virol. 2018 Jul 31;92(16):e00145-18. doi: 10.1128/JVI.00145-18 (PMC6069186; doi:10.1128/JVI.00145-18)
Supplement: Supplemental material [file JVI.00145-18_zjv016183759s1.pdf]

Figure S1. **Phylogeny of exogenous and endogenous circovirus Rep sequences.**

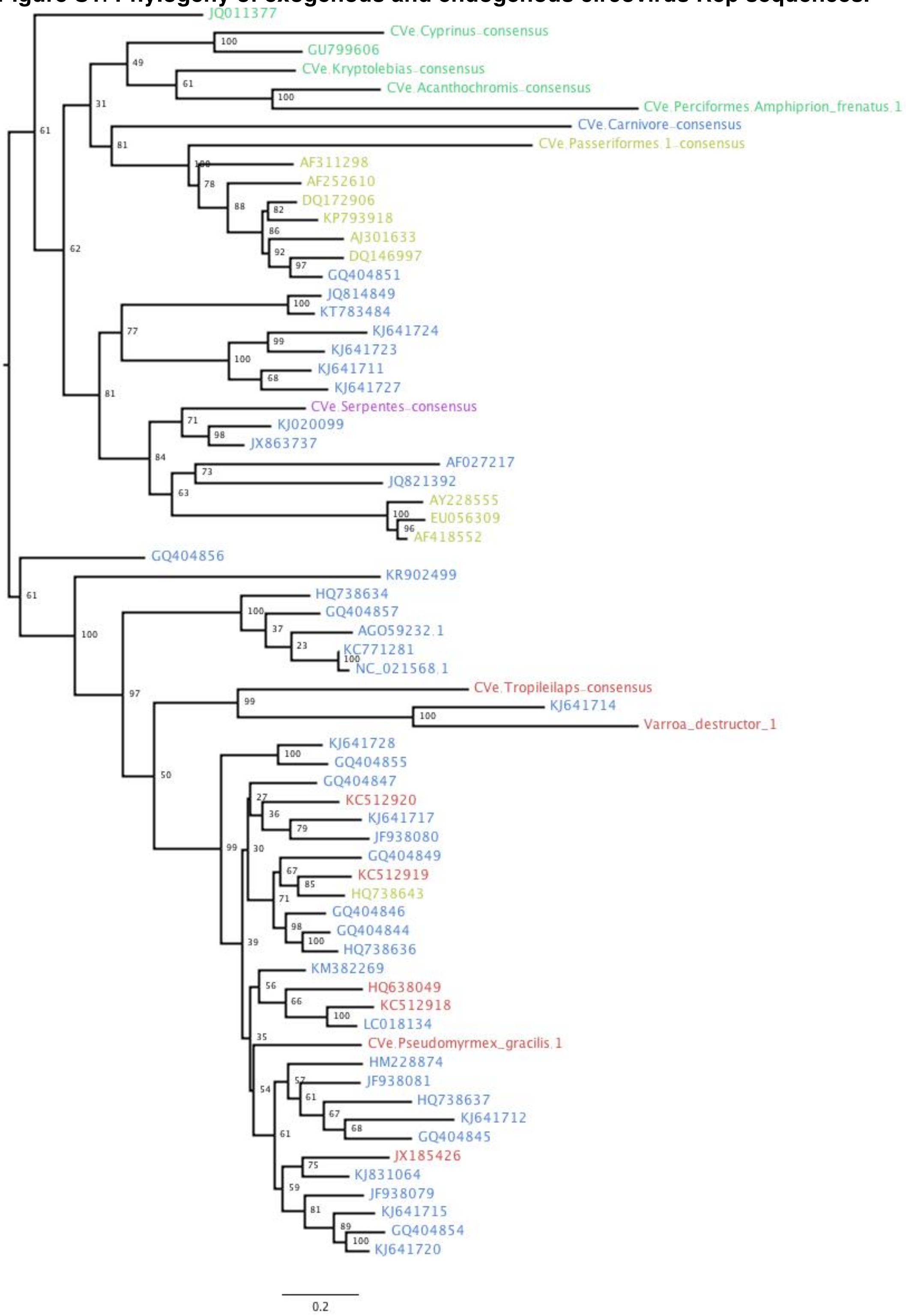

Figure S1. Maximum-likelihood phylogeny reconstructed from an alignment of replication-associated protein (Rep) sequences. The tree is mid-point rooted, asterisks indicate nodes with >70% bootstrap support. Scale bar indicates evolutionary distance in substitutions per site. Sequences derived from metagenomic samples are indicated by colored circles. Taxa names are shown as accession numbers for sequences derived from viruses and metagenomic samples, and element IDs for CVe.

Table S1. Circovirus and Cyclovirus reference sequences

Accession #	Virus name	Citation*
**Circovirus**		
NC_001944	Beak and feather disease virus	(1)
NC_002361	Columbid circovirus	(2)
NC_003410	Canary circovirus	(3)
NC_008033	Starling circovirus (StCV)	(4)
NC_008375	Raven circovirus	(5)
NC_008521	Gull circovirus	(6)
NC_008522	Finch circovirus	(6)
NC_026945	Zebra finch circovirus	(7)
NC_007220	Duck circovirus	(8)
NC_025247	Swan circovirus	(9)
NC_015399	Barbel circovirus	(10)
NC_025246	Wels catfish circovirus	(11)
NC_001792	Porcine circovirus 1	(12)
NC_005148	Porcine circovirus 2	(12)
NC_020904	Canine circovirus 1	(13)
NC_023885	Mink circovirus	(14)
NC_028045	Mexican free-tailed bat circovirus	(15)
NC_031753	Porcine circovirus 3	(16)
GQ404851	Chimpanzee stool avian-like circovirus Chimp17	(17)
JQ814849	Bat associated circovirus 3	(18)
KJ641711	Bat associated circovirus 8	(19)
KT783484	Bat associated circovirus 4	(20)
KJ641724	Bat associated circovirus 6	(19)
KJ641711	Bat associated circovirus 8	(19)
KJ641723	Bat associated circovirus 7	(19)
**Cyclovirus**		
KR902499	Cyclovirus Equ1	(30)
HQ738634	Cyclovirus PKbeef23	(21)
GQ404857	Cyclovirus TN25	(17)
KF031471	Cyclovirus VN isolate cs1	(22)
NC_021568	Human cyclovirus VS5700009	(23)
KJ641714	Bat circovirus isolate BtRp-CV-14/GD2012	(18)
KJ641728	Bat associated cyclovirus 13 isolate BtPa-CV-2/NX2013	(18)
GQ404855	Cyclovirus NG14	(17)
GQ404847	Cyclovirus PK5510	(17)
KC512920	Dragonfly cyclovirus 8	(24)
KJ641717	Bat associated cyclovirus 11 isolate BtMspp.-CV/GD2012	(18)
JF938080	Bat circovirus ZS/China/2011 isolate YN-BtCV-3	(25)
GQ404849	Cyclovirus Chimp11	(17)
KC512919	Dragonfly cyclovirus 7	(24)
HQ738643	CyCV-NG-chicken-8	(21)
GQ404846	Cyclovirus PK5222	(17)
GQ404844	Cyclovirus PK5006	(17)
HQ738636	Cyclovirus PKgoat11	(21)
KM382269	Bat circovirus POA/2012/II	(20)
HQ638049	Dragonfly cyclovirus isolate DfCyV-A9	(26)
KC512918	Dragonfly cyclovirus 6	(24)
LC018134	Cyclovirus TsCyV-1_JP-NUBS-2014	(27)
HM228874	Bat cyclovirus GF-4c	(28)
JF938081	Bat associated cyclovirus 3 isolate YN-BtCV-4	(25)
HQ738637	Cyclovirus bat/USA/2009	(21)
KJ641712	Bat associated cyclovirus 6	(18)
GQ404845	Cyclovirus PK5034	(17)
JX185426	Dragonfly cyclovirus 5	(29)
KJ831064	Cyclovirus SL-108277	(18)
JF938079	Bat associated cyclovirus 2 isolate YN-BtCV-2	(25)
KJ641715	Bat associated cyclovirus 8 isolate BtRp-CV-52/GD2012	(18)
GQ404854	Cyclovirus NG12	(17)
KJ641720	Bat associated cyclovirus 9 isolate BtTp-CV-2/GX2012	(18)
**Unclassified**		
GQ404856	Human stool-associated circular virus NG13	(17)

Footnote: *First report, or review describing history of discovery.

References

1. **Niagro FD, Forsthoefel AN, Lawther RP, Kamalanathan L, Ritchie BW, Latimer KS, Lukert PD.** 1998. Beak and feather disease virus and porcine circovirus genomes: intermediates between the geminiviruses and plant circoviruses. Arch Virol **143:**1723-1744.
2. **Mankertz A, Hattermann K, Ehlers B, Soike D.** 2000. Cloning and sequencing of columbid circovirus (coCV), a new circovirus from pigeons. Arch Virol **145:**2469-2479.
3. **Todd D, Weston J, Ball NW, Borghmans BJ, Smyth JA, Gelmini L, Lavazza A.** 2001. Nucleotide sequence-based identification of a novel circovirus of canaries. Avian Pathol **30:**321-325.
4. **Johne R, Fernandez-de-Luco D, Hofle U, Muller H.** 2006. Genome of a novel circovirus of starlings, amplified by multiply primed rolling-circle amplification. J Gen Virol **87:**1189-1195.
5. **Stewart ME, Perry R, Raidal SR.** 2006. Identification of a novel circovirus in Australian ravens (Corvus coronoides) with feather disease. Avian Pathol **35:**86-92.
6. **Todd D, Scott AN, Fringuelli E, Shivraprasad HL, Gavier-Widen D, Smyth JA.** 2007. Molecular characterization of novel circoviruses from finch and gull. Avian Pathol **36:**75-81.
7. **Rinder M, Schmitz A, Peschel A, Korbel R.** 2015. Complete genome sequence of a novel circovirus from zebra finch. Genome Announc **3**.
8. **Banda A, Galloway-Haskins RI, Sandhu TS, Schat KA.** 2007. Genetic analysis of a duck circovirus detected in commercial Pekin ducks in New York. Avian Dis **51:**90-95.
9. **Halami MY, Nieper H, Muller H, Johne R.** 2008. Detection of a novel circovirus in mute swans (Cygnus olor) by using nested broad-spectrum PCR. Virus Res **132:**208-212.
10. **Lorincz M, Csagola A, Farkas SL, Szekely C, Tuboly T.** 2011. First detection and analysis of a fish circovirus. J Gen Virol **92:**1817-1821.
11. **Lorincz M, Dan A, Lang M, Csaba G, Toth AG, Szekely C, Csagola A, Tuboly T.** 2012. Novel circovirus in European catfish (Silurus glanis). Arch Virol **157:**1173-1176.
12. **Ellis J.** 2014. Porcine circovirus: a historical perspective. Vet Pathol **51:**315-327.
13. **Li L, McGraw S, Zhu K, Leutenegger CM, Marks SL, Kubiski S, Gaffney P, Dela Cruz FN, Jr., Wang C, Delwart E, Pesavento PA.** 2013. Circovirus in tissues of dogs with vasculitis and hemorrhage. Emerg Infect Dis **19:**534-541.
14. **Lian H, Liu Y, Li N, Wang Y, Zhang S, Hu R.** 2014. Novel circovirus from mink, China. Emerg Infect Dis **20:**1548-1550.
15. **Lima FE, Cibulski SP, Dall Bello AG, Mayer FQ, Witt AA, Roehe PM, d'Azevedo PA.** 2015. A Novel Chiropteran Circovirus Genome Recovered from a Brazilian Insectivorous Bat Species. Genome Announc **3**.
16. **Palinski R, Pineyro P, Shang P, Yuan F, Guo R, Fang Y, Byers E, Hause BM.** 2017. A Novel Porcine Circovirus Distantly Related to Known Circoviruses Is Associated with Porcine Dermatitis and Nephropathy Syndrome and Reproductive Failure. J Virol **91**.

17. **Li L, Kapoor A, Slikas B, Bamidele OS, Wang C, Shaukat S, Masroor MA, Wilson ML, Ndjango JB, Peeters M, Gross-Camp ND, Muller MN, Hahn BH, Wolfe ND, Triki H, Bartkus J, Zaidi SZ, Delwart E.** 2010. Multiple diverse circoviruses infect farm animals and are commonly found in human and chimpanzee feces. J Virol **84:**1674-1682.
18. **Wu Z, Ren X, Yang L, Hu Y, Yang J, He G, Zhang J, Dong J, Sun L, Du J, Liu L, Xue Y, Wang J, Yang F, Zhang S, Jin Q.** 2012. Virome analysis for identification of novel mammalian viruses in bat species from Chinese provinces. J Virol **86:**10999-11012.
19. **Wu Z, Yang L, Ren X, He G, Zhang J, Yang J, Qian Z, Dong J, Sun L, Zhu Y, Du J, Yang F, Zhang S, Jin Q.** 2016. Deciphering the bat virome catalog to better understand the ecological diversity of bat viruses and the bat origin of emerging infectious diseases. Isme j **10:**609-620.
20. **Lima FE, Cibulski SP, Dos Santos HF, Teixeira TF, Varela AP, Roehe PM, Delwart E, Franco AC.** 2015. Genomic characterization of novel circular ssDNA viruses from insectivorous bats in Southern Brazil. PLoS One **10:**e0118070.
21. **Li L, Shan T, Soji OB, Alam MM, Kunz TH, Zaidi SZ, Delwart E.** 2011. Possible cross-species transmission of circoviruses and cycloviruses among farm animals. J Gen Virol **92:**768-772.
22. **Tan le V, van Doorn HR, Nghia HD, Chau TT, Tu le TP, de Vries M, Canuti M, Deijs M, Jebbink MF, Baker S, Bryant JE, Tham NT, NT BK, Boni MF, Loi TQ, Phuong le T, Verhoeven JT, Crusat M, Jeeninga RE, Schultsz C, Chau NV, Hien TT, van der Hoek L, Farrar J, de Jong MD.** 2013. Identification of a new cyclovirus in cerebrospinal fluid of patients with acute central nervous system infections. MBio **4:**e00231-00213.
23. **Smits SL, Zijlstra EE, van Hellemond JJ, Schapendonk CM, Bodewes R, Schurch AC, Haagmans BL, Osterhaus AD.** 2013. Novel cyclovirus in human cerebrospinal fluid, Malawi, 2010-2011. Emerg Infect Dis **19**.
24. **Dayaram A, Potter KA, Moline AB, Rosenstein DD, Marinov M, Thomas JE, Breitbart M, Rosario K, Arguello-Astorga GR, Varsani A.** 2013. High global diversity of cycloviruses amongst dragonflies. J Gen Virol **94:**1827-1840.
25. **Ge X, Li J, Peng C, Wu L, Yang X, Wu Y, Zhang Y, Shi Z.** 2011. Genetic diversity of novel circular ssDNA viruses in bats in China. J Gen Virol **92:**2646-2653.
26. **Rosario K, Marinov M, Stainton D, Kraberger S, Wiltshire EJ, Collings DA, Walters M, Martin DP, Breitbart M, Varsani A.** 2011. Dragonfly cyclovirus, a novel single-stranded DNA virus discovered in dragonflies (Odonata: Anisoptera). J Gen Virol **92:**1302-1308.
27. **Sato G, Kawashima T, Kiuchi M, Tohya Y.** 2015. Novel cyclovirus detected in the intestinal contents of Taiwan squirrels (Callosciurus erythraeus thaiwanensis). Virus Genes **51:**148-151.
28. **Li L, Victoria JG, Wang C, Jones M, Fellers GM, Kunz TH, Delwart E.** 2010. Bat guano virome: predominance of dietary viruses from insects and plants plus novel mammalian viruses. J Virol **84:**6955-6965.
29. **Rosario K, Dayaram A, Marinov M, Ware J, Kraberger S, Stainton D, Breitbart M, Varsani A.** 2012. Diverse circular ssDNA viruses discovered in dragonflies (Odonata: Epiprocta). J Gen Virol **93:**2668-2681.
30. **Li L, Giannitti F, Low J, Keyes C, Ullmann LS, Deng X, Aleman M, Pesavento PA, Pusterla N, Delwart E.** 2015. Exploring the virome of diseased horses. J Gen Virol **96:**2721-2733.
